# Supplementary material for: Can green credit policies improve the digital transformation of heavily polluting enterprises: A quasi-natural experiment based on difference-in-differences
Source: PLoS One. 2024 Aug 29;19(8):e0307722. doi: 10.1371/journal.pone.0307722 (PMC11361581; doi:10.1371/journal.pone.0307722)
Supplement: Attachment — Submitted filename: Response to Reviewer2.docx [file pone.0307722.s002.docx]

Dear  Reviewer

Thanks for the your valuable comments, we have carefully read and understood your revision suggestions. Here, we are deeply grateful for every question you have raised, and have revised accordingly according to your comments. We believe this will greatly improve the quality of our papers.Responses to each question are indicated in red font as follows:

Reviewer #1: In the abstract section, both R&D investment and executives' financial background weaken the inhibitory effect, but only the specific impact of R&D investment is described in depth. Why not explain the impact of executives' financial backgrounds? It is suggested to supplement the detailed influence of executives' financial backgrounds or delete the specific expression of the impact of R&D investment.

Response #1：Thank you very much for the lack of a description of the weakening effect of the executive's financial background in the abstract of this article, and we have added a description of its specific impact in the abstract to make the summary more rigorous. Specifically, " Enterprises with senior financial expertise have a deeper understanding of financial feasibility and benefit analysis, and are more receptive to the high-risk investment of digital transformation, while their financial network resources can help broaden financing channels, reduce financing constraints, and further reduce the financial difficulty of digital transformation.".

Reviewer #2： In the introductory section, on page 2 of the manuscript, the first paragraph proposes that environmental protection and digital transformation are the two major themes leading the future development, but only the role of digital transformation on corporate strategy is introduced later, and the impact of environmental protection on corporate strategy is not introduced separately. It is suggested that "on the other hand" should be followed by a description of the importance of environmental protection for manufacturing enterprises. The relevant descriptions of existing green credit policies are suggested to be moved after the introduction of the two themes and introduced as a policy tool linking the two themes. The symbols cited in pages 2-3 of the manuscript are incorrectly marked and should be replaced with superscripts.

Response #2：Thank you very much for your valuable comments in the introduction of this article, and I very much agree with and thank you very much for your comments, which greatly improves the citation quality of this article. We have already emphasized the importance of environmental protection for manufacturing companies after the " "on the other hand" " in the introduction, while also moving the description of green credit policy back and introducing it as a policy tool that bridges the two themes. Specifically, "On the other hand, environmental protection is of great importance in today's global economy. Manufacturing companies must comply with increasingly stringent environmental regulations and standards, which is not only a social responsibility for enterprises, but also an important way to achieve sustainable development. Environmental requirements are driving companies to innovate in technology and business models, and to explore new development opportunities. Through R&D and application of environmental protection technologies, enterprises can develop new products and services, and open up new markets, which can not only enable the manufacturing industry to meet the regulatory requirements of the green environment and social expectations, but also improve resource utilization efficiency, reduce operational risks, enhance market competitiveness, and explore innovation and development opportunities. Driven by digitalization and environmental protection, manufacturing enterprises should integrate environmental protection into their strategic planning, promote green transformation, and achieve a win-win situation of economic and environmental benefits.”

In addition, I have replaced all of the symbols cited the manuscript with superscripts.

Reviewer #3：In the research design section; on page 7 of the manuscript, please re-edit the three model formulas according to the publication standards of the journal, especially formula (3) is deformed. On page 8 of the manuscript, there are errors and repetitions in the description of assignment in the explained variable part of variable selection. There is no mention of how Digit_text calculates, and readers cannot understand the formation process of the explained variable according to the description. On page 9 of the manuscript, there are many repetitions and errors in the wording of the control variables; some variable names in Table 1 are not translated properly, such as "two jobs in one".

Response #3：First of all, thank you very much for raising the problem about the distortion of the formula model format in the article, and we will correct it according to the requirements of the journal, as follows：

|  | (1) | |
| --- | --- | --- |
|  \| (2);  \| (3) | |  |

Secondly, we have explained in detail the formation process of the explanatory variable (Digit) in the manuscript, and the calculation process of the Digit_text has been supplemented. The details are as follows：

“The explained variable in this paper is the level of digital transformation of the enterprise, referring to the research results of Chen et al. (2021)^［23］^ : Based on the statistics of 99 digital-related word frequencies in four dimensions: digital technology application, Internet business model, intelligent manufacturing, and modern information system, the digital transformation index of manufacturing enterprises was constructed by using text analysis method and expert scoring method. First, use text analytics to construct Digit_text variables. The first step is to collect the annual reports of listed companies in the manufacturing industry from 2008 to 2022 and convert them into text format, and then extract the text of the business analysis part through Python. The second step is to extract a certain number of samples of enterprises that have been successful in digital transformation through manual judgment. In the third step, the selected samples were processed by word segmentation and word frequency statistics to screen out high-frequency words related to digital transformation, which can be divided into four dimensions: digital technology application, Internet business model, intelligent manufacturing and modern information system, which suggests that we can construct the digital transformation index of enterprises from four dimensions (see Table 1). In the fourth step, based on the words formed in the third step, the text before and after is extracted from the total sample of listed companies, and the text combinations with high frequency are found. The fifth step is to supplement the keywords on the basis of the existing literature to form the final word segmentation dictionary. In the sixth step, based on the self-built word segmentation dictionary, the Jieba function is used to segment all samples, and the number of keyword disclosures is counted from four aspects: digital technology application, Internet business model, intelligent manufacturing and modern information system, so as to reflect the degree of transformation of the enterprise in all aspects. On this basis, the word frequency data was standardized, and the entropy method was used to determine the weight of each index, and finally the Digit_text index was obtained.”

Table 1 Enterprise digital transformation index construction and keyword selection

| Dimension | Categorical terms | Combinations of text that appear more frequently | Word Segmentation Dictionary |
| --- | --- | --- | --- |
| Digital  technology  applications | Data, numbers,  digitization | Data Management, Data Mining, Data  Networks, Data Platforms, Data Centers, Data Science, Digital Control, Digital  Technology, Digital Communications,  Digital Networks, Digital Intelligence,  Digital Terminals, Digital Marketing  Digitalization | Data Management, Data Mining, Data Network, Data Platform, Data Center, Data Science, Digital Control, Digital Technology,  Digital Communication, Digital Network, Digital Intelligence,  Digital Terminal, Digital Marketing, Digitalization, Big Data,  Cloud Computing, Cloud IT, Cloud Ecology, Cloud Service, Cloud Platform, Blockchain, Internet of Things, Machine Learning |
| Internet  business  model | Internet,  e-commerce | Mobile Internet, Industrial Internet,  Industrial Internet, Internet Solutions,  Internet Technology, Internet Thinking, Internet Action, Internet Business,  Internet Mobile, Internet Application,  Internet Marketing, Internet Strategy,  Internet Platform, Internet Model,  Internet Business Model, Internet  Ecology, E-commerce, E-commerce | Mobile Internet, Industrial Internet, Industrial Internet,  Internet Solutions, Internet Technology, Internet Thinking,  Internet Action, Internet Business, Internet Mobile, Internet Application, Internet Marketing, Internet Strategy, Internet  Platform, Internet model, Internet business model Internet  ecology, e-commerce, e-commerce, Internet, "Internet +",  online Offline, online-to-offline, online, and offline、O2O、  B2B、C2C、B2C、C2B |
| Smart  manufacturing | Intelligent,  intelligent, automatic,  numerical control,  integration,  integration | Artificial intelligence, high-end  intelligence, industrial intelligence,  mobile intelligence, intelligent control,  intelligent terminal, intelligent mobile,  intelligent management, intelligent  factory, intelligent logistics, intelligent manufacturing, intelligent warehousing, intelligent technology, intelligent  equipment, intelligent production,  intelligent networking, intelligent  system, intelligence, automatic control,  automatic monitoring, automatic  monitoring, automatic detection,  automatic production, numerical  control, integration, integration,  integrated solution, integrated control,  integrated system | Artificial Intelligence, High-end Intelligence, Industrial  Intelligence, Mobile Intelligence, Intelligent Control,  Intelligent Terminal, Intelligent Mobile, Intelligent  Management, Intelligent Factory, Intelligent Logistics,  Intelligent Manufacturing, Intelligent Warehousing, IntelligentTechnology, Intelligent Equipment, Intelligent Production,  Intelligent Networking, Intelligent System, Intelligent,  Automatic Control, Automatic Monitoring, Automatic  Monitoring, Automatic Detection, Automatic Production,  CNC, Integration, Integration, Integrated Solution, Integrated Control, Integrated System, Industrial Cloud, Future Factory,Intelligent Fault Diagnosis, Life Cycle Management,  Manufacturing Execution Systems, Virtualization, Virtual  Manufacturing |
| Modern  information  systems | Information,  informatization, networking | Information sharing, information  management, information integration,  information software, information  systems, information networks,  information terminals, information  centers, informatization, networking | Information sharing, information management, information  integration, information software, information systems,  information networks, information terminals, information  centers, informatization, networking, industrial information,  industrial communication |

Finally, we have changed the wording of the description of the control variables in the manuscript and correctly translated the names of the variables in Table 1 of the variables. For example, translate "dual" correctly as:“The chairman and general manager are the same person as 1, otherwise 0”

Reviewer #4： In the empirical results and analysis section, on page 10 of the manuscript, the results reported in Table 3 are incorrect, possibly missing the lower part of the regression results. On pages 14-15 of the manuscript, the moderating effect of R&D investment and executives with financial background is to test hypothesis 2 and hypothesis 3 respectively, which are incorrectly written as hypothesis 3 and hypothesis 4 in the manuscript.

Response #4：We have corrected the regression results and text analysis sections in Table 3. At the same time, the moderating effects of R&D investment and executives' financial background in the manuscript are corrected to test hypothesis 2 and hypothesis 3, respectively.

Reviewer #5： In general, it is not necessary to write the full name of the author in the cited literature in the paper, but the surname can be written. The full name is used in many places in the paper, and it is suggested to modify it. For example, in line 2 of page 11 of the manuscript, "Zhang Xiaoxiao and Hu Jinyan(2022)" should be changed to "Zhang and Hu (2022)".

Response #5：Thank you very much for your valuable comments! We have corrected the format of the citations in the manuscript so that only the author's last name is written.

Reviewer #6：On page 16 of the manuscript, the acknowledgments should be placed at the end of the paper.

Response #6：Thank you for your valuable comments, taking into account the journal editor's request that "no funding information about the manuscript appear anywhere in the text". Therefore, I have deleted the acknowledgments in the manuscript.

Reviewer #7：On page 17 of the manuscript, section of conclusions and suggestions, the first paragraph of the conclusion is repeated and difficult to understand, please re-describe the research content in concise language; the three paragraphs of the research conclusion can be combined into one paragraph, in which "the Guidelines" in the second and third paragraphs are proposed to be changed to "the green credit policy". Policy suggestions should be put forward from the perspectives of different subjects. The current policy suggestions are all from the government’s perspective and lack enterprises’ subjectivity.

Response #7:Thank you again for your valuable comments! The first paragraph of the conclusions in the manuscript has been refined. The research content of this paper is condensed, and the three main findings of the research conclusions are synthesized into one paragraph. At the same time, the "guidelines" in the second and third paragraphs of the original text are proposed to be changed to "green credit policies". Finally, the policy recommendations in the manuscript were revised and improved, and suggestions were made from the perspectives of the government and enterprises.

The details are as follows:“Based on the Green Credit Guidelines issued in 2012, this paper selects China's manufacturing A-share listed companies from 2008 to 2022 as the research sample. Based on the existing research, this paper uses the difference-in-differences method to investigate and evaluate the impact of green credit policy on the digital transformation of heavily polluting enterprises. The research results show that: Firstly, the green credit policy, represented by “the Green Credit Guidelines”, has a significant inhibitory effect on the digital transformation of heavily polluting enterprises. Secondly, from the perspective of regulating mechanism, the R&D investment of enterprises and the financial background of executives weaken the inhibitory effect of “the green credit policy” on the digital transformation of heavily polluting enterprises, and the inhibitory effect exerted by the policy is more obvious when the R&D investment is low, but the inhibitory effect brought about by the policy decreases gradually with the increase of R&D investment of enterprises, i.e., the R&D investment of enterprises and “the green credit policy” have an obvious That is, enterprise R&D investment and “the Green Credit Guidelines” have an obvious substitution relationship in influencing the digital transformation of heavy pollution enterprises. Thirdly, “the Green Credit Guidelines” has a significantly stronger inhibitory effect on the digital transformation of non-SOE heavy polluters than that of SOEs; it has a significant inhibitory effect on the digital transformation of heavy polluters that do not hold shares in a bank, while the effect on heavy polluters that hold shares in a bank is insignificant.

Based on the above conclusions, this paper puts forward the following policy recommendations from the perspectives of government and enterprises.

On the one hand, the government should launch a special digital transformation loan program to provide heavily polluting enterprises with preferential conditions such as low interest rates and extended repayment periods, so as to ensure that they receive adequate financial support in the process of digital transformation. At the same time, the government should encourage enterprises to increase R&D investment, such as through tax incentives and scientific research funding support, to encourage enterprises to increase R&D investment in the field of digitalization. Flexibly adjust the green credit conditions according to the level of enterprise R&D investment, and provide more flexible credit support for enterprises with low R&D investment. In addition, the government should implement differentiated green credit policies. Formulate differentiated policies according to the nature and shareholding of enterprises, and promote close cooperation between non-state-owned enterprises and non-bank shares and financial institutions to ensure that these enterprises can obtain favorable financial support. On the other hand, enterprises should actively apply for the government's digital transformation loan program to take advantage of low interest rates and flexible repayment terms to reduce financing pressure and ensure the funds needed for digital upgrading. At the same time, enterprises should increase R&D investment and increase digital technology R&D and innovation activities to improve their competitiveness. In addition, enterprises should pay attention to financial literacy training such as digital literacy of senior executives, and encourage enterprises to participate in training programs to enhance their understanding and support for digital transformation. Finally, companies should optimize their financing structures and strengthen financial cooperation. Specifically, non-state-owned enterprises should explore flexible financing methods and establish close cooperation with financial institutions to obtain favorable financial support. Companies with bank stakes should optimize their financing structures and leverage their banking relationships to obtain better financing conditions to support digital transformation.”

Reviewer #8：There are a lot of grammatical and lexical errors in the paper, so it is recommended to polish it carefully.

Response #8：We have carefully polished the manuscript and carefully corrected any grammatical and lexical errors that occurred. Thank you for your careful review of this article, which greatly enhances the quality and value of this article, and thank you again for your hard work on this article.
